# The standard of integrity may be useful when assessing arguments over qualitative review methods: The case of the Joanna Briggs Institute's rebuttal of a fundamental critique

**DOI:** 10.1111/nin.12465

**Published:** 2021-09-25

**Authors:** Marielle de Vaal, Peter Andrew Tamás

**Affiliations:** ^1^ Department of Agrotechnology and Food Sciences Wageningen University & Research Food Quality and Design Wageningen The Netherlands; ^2^ Department of Plant Sciences, Division of Mathematical and Statistical Methods–Biometris Wageningen University & Research Wageningen The Netherlands

**Keywords:** argument analysis, argument mapping, domain analysis, meta‐synthesis, methodology, qualitative methods, quality assessment, systematic reviews and meta‐analyses

## Abstract

One challenge for those reading methodological debates in low consensus fields is determining the outcome when participants do not share standards. When parties to a debate do not agree on the standards to be used in assessing their arguments (i.e., quality), it may be useful to ask first if parties’ contributions meet their own expectations (i.e., integrity). Most protocols for review of qualitative research specify some form of quality assessment. These protocols normally require some test of internal coherence. Coherence is also relevant when describing the match between a rebuttal and the argument it answers. In 2019, *Nursing Inquiry* published a critique and rebuttal of the methods used by the Joanna Briggs Institute. In this essay, we attempted to use the Joanna Briggs Institute's own quality assessment standards to assess their rebuttal of this fundamental critique. We found it possible to use the Joanna Briggs Institute's own quality assessment standards to assess this rebuttal, and we found that JBI's rebuttal did not meet their own standards.

## 1 INTRODUCTION

In 2019, *Nursing Inquiry* published an exchange between (Bergdahl, 2019) (hereafter Bergdahl) who wrote a piece quite critical of meta‐aggregation as used by the Joanna Briggs Institute, and (Lockwood et al., 2019) (hereafter JBI, as this is clearly an institutional response) who attempted to rebut Bergdahl. Bergdahl's critique is a clearly stated turn in long‐standing efforts to find methods that permit comparison between, and synthesis of, results across highly heterogeneous qualitative studies. This history, well introduced by Thorne (2019), provides the standards used by Bergdahl to ask fundamental questions about the work done by JBI. The logic behind Bergdahl's intervention is that of external critique: the work done by JBI should meet standards endorsed by Bergdahl. On these external grounds, Bergdahl found the work done by JBI to be severely deficient. The defence mounted by JBI, demonstrating that the heterogeneity that characterizes qualitative inquiry extends to those who review, referenced their own standards in finding their work to be sound. The resulting impasse is not helpful to those who read such exchanges for guidance.

Our purpose in this essay was to find a means to help adjudicate debates over review methods when parties do not agree on standards. We accept that there are many different understandings of quality (Moss et al., 2009) that these different understandings of quality inform contributions to debates over qualitative review methods, and that parties may object to having others’ standards imposed upon them. Our hope was to find an expectation that was both reasonable and workable to hold before external standards. The expectation that we adopted is that parties speak with integrity, which we understand as walking our own talk. The definition and operationalization of integrity we adopted, descends from a simple understanding of coherentism that takes more interest in hypocrisy than, as is more typical, in the extent to which statements “hang together” (Douven & Meijs, 2007) or are “nonself‐contradictory” (Madill et al., 2000). Where the latter contributions tend to be more tied to discussion of the relationship between coherence and correspondence in the discussion of the conditions required for claims to be recognized as truths (Olsson & Schubert, 2007), our interest is more mundane. We just want to be able to comment on the merits of the theory subsequent to which empirical results are found. Our interest in the ability to use results to comment on their authorizing theory descends from our assumption of a fairly naïve convergent realism handily described by Kuukkanen (2007). Specifically, we assume that there is an independent real out there for whose description we have not yet developed adequate theory or methods. As such, while we at this point may suffer from the confusions introduced by a diversity of flawed theories and methods, disciplined operationalization of theories and methods over time will iteratively allow us to toss those theories and methods that are consistently found worst. Integrity is critical when working with these assumptions as far as hypocrisy destroys the ability to make valid inferences from results to theory. The perhaps unwarranted expectation that we have in this essay, is that we may reasonably expect those who review qualitative research in low consensus domains where such integrity is particularly relevant, and who expect such integrity of the research they review, to demonstrate the same integrity in their own conduct.

The methodological purpose of this essay is, thus, to query the extent to which integrity is a workable criterion for examining debates over the methods used in the qualitative evidence review. The first step required in such an effort is to identify appropriate standards. These standards will be expectations declared by a party to a debate, that are relevant for consideration of their contributions to that debate. In the exchange between Bergdahl and JBI, both advocate their own review methods. JBI's response is institutional and JBI does declare standards that they expect of the reports they review. Therefore, it is reasonable to ask if JBI's institutionally declared standards are appropriate for consideration of their practice when defending their methods. Since there is neither an equivalent set of standards for Bergdahl, nor is it reasonable to assume that Bergdahl would accept JBI's (respecting the possibility of heterogeneity), this essay will only consider JBI's response to Bergdahl's critique.

The JBI does not clearly state standards for the assessment of their engagement in debates over methods. However, they do declare how review of qualitative studies ought to be done. One of the steps required within review conducted according to the protocols created by JBI and others who support review of qualitative studies (e.g., those suggested by Carroll et al., 2012; Dixon‐Woods et al., 2004, 2006; Fossey et al., 2002; Kmet et al., 2004; Pawson et al., 2003; Spencer et al., 2003; Tong et al., 2012), is an assessment of the quality of each study being considered. For this, JBI has developed a quality assessment checklist (Joanna Briggs Institute, 2020). Four of the ten items on their list test for external standards (reflexivity, instrument effects, respect for respondent voice, and compliance with ethics standards), and the remaining six items, test whether the components of a study fit together. These internal items ask the reviewer to determine congruence between theory, methodology, objectives, questions, data collection methods, representation of data, analysis methods, interpretation, and conclusions.

Testing congruence requires three capacities. The first capacity entails the correct recognition of analytically relevant features. The reviewer must, for example, be able to recognize and correctly characterize both research questions and data collection methods. The second capacity concerns correct recognition of relationships between features. The reviewer must, continuing the example, recognize the extent to which the collected data matches the variables specified in the question. The third capacity entails a correct assessment of the adequacy of each of the relevant features. The reviewer must be able to determine if the results put forward have any backing at all, and if so, the appropriateness of that backing.

The capacities required to test congruence within qualitative studies, as endorsed by JBI, have direct analogues for the assessment of their rebuttal of critiques, such as that presented by Bergdahl: (1) JBI must be able to correctly detect the analytically relevant aspects of a critique, indicated by correct, and explicit recognition of the definitions given key terms; (2) JBI must be able to correctly recognize the content of a critique, indicated by rebuttals that recognize and address all relevant aspects of a critique; and (3) JBI must be able to provide an adequate response, indicated by rebuttals that are accompanied by some form of recognizably appropriate backing. These specific expectations that, together, operationalize congruence as applied to JBI's refutation of a critique of their methodology support the following set of substantive research questions:

- To what extent does JBI recognize Bergdahl's definitions of key terms?
- To what extent does the content of JBI's rebuttals match the content of the matching critiques?
- To what extent do JBI's rebuttals reference recognizable backing?

## 2 METHODS

JBI's response to Bergdahl's critique was tested using three qualitative analysis methods: domain, thematic content, and argument analysis. The two authors worked on the texts simultaneously but independently. The authors used a formative approach to reliability, in which we consulted on decisions when either of us was at all uncertain, rather than summative testing of inter‐rater reliability. This formative approach was indicated by the exploratory character of our task, the nature of the decisions we were making (relational, rather than taxonomic), and the small data set with which we were working with.

Our first step was complete immersion with the texts of Bergdahl and JBI, to become familiar with both texts. Prior experience indicated that we could not assume that either Bergdahl or JBI was well‐structured texts, in which all material relevant to any given point was located within the same paragraph. Therefore, both papers were printed on hardcopy and read several times to identify claims and the text relevant to each. This same process of immersion and identification of related material was then undertaken in the qualitative data analysis software Atlas.ti version 8. The results of the two efforts were then compared and reconciled by the first author of the essay. Once complete, results were reviewed by the second author. Comments made by the second author were discussed with the first author until consensus was reached on the relation between quotations from the texts of JBI and Bergdahl. Throughout analysis and review, both authors deliberately chose to be strongly biased in favor of finding that JBI's arguments met their own standards. This charitable bias was adopted as it is consistent with our own strategy for handling missing data in review: we assume that authors are well‐intentioned and competent until proven otherwise.

Our rigor, the quality of being very exact, careful, thorough, and precise with the data (Cypress, 2017), was strengthened in part through deliberate reflexivity: insight into our own biases and rationales for decision‐making during the analyses (Johnson et al., 2020; Maher et al., 2018). Reflexivity was strengthened by initial clear statement of expectations constituted through critical debate between the authors, interactive immersion in and interactions with the texts of Bergdahl and JBI, both as sources of empirical information as inspiration to trigger the application of imaginative insight during the attempts to understand the texts. Following on these analyses were planned deliberately to make biases explicit and to subject them to debate. Potential findings were discussed and continuously reflected upon: throughout the analysis, those data whose categorization was even slightly uncertain were set aside for discussion to eliminate the opportunity for one researcher's biases to silently inform recognition of our data. Results were reported as completely, accurately, and objectively as possible. These processes were supported by the combination of traditional reading, coding, and linking of the texts of Bergdahl and JBI, and the use of the digital analysis software package Atlas.ti to read, code, and link the texts. This combination of traditional and digital analyses provided the breadth required for immediate identification of links between disparate segments of text and excellent data management and retrieval facilities, which supported the rigorous analyses and reporting of the findings from all analysis methods.

### 2.1 Domain analysis

Our efforts to determine the extent to which JBI correctly recognized Bergdahl's definitions of key terms were guided by cultural domain analysis. Domain analysis was selected as this method was created for the specific purpose of inductively determining how participants partition their life worlds (Borgatti, 1994). This was tested by determining the match (by specification or rejection) between JBI's and Bergdahl's explicit definition of the following terms selected for their salience: meta‐aggregation, meta‐synthesis, and qualitative sciences. A lack of match on any one of these key terms was taken as an indication of JBI's failure to correctly recognize Bergdahl's definitions of key terms. To ensure that human coding did not miss any instance of these key terms, domain analysis started with automated coding within Atlas.ti that separately targeted the search terms *meta‐aggregation*, *aggregation*, *meta‐synthesis*, *synthesis*, *social science(s)*, *qualitative science(s)*, and *qualitative research*. Passages in which these terms were found were examined to see whether the surrounding discussion displayed the authors’ own or adopted definitions of the term. Passages that were found to display the authors’ own or adopted definitions were coded and those passages were linked to the term defined.

All passages presenting both texts’ definitions of the terms meta‐aggregation, meta‐synthesis, and qualitative sciences were extracted and losslessly aggregated. This produced a complete account of the content associated with each term for both. The content associated with meta‐aggregation, meta‐synthesis, and qualitative sciences were then compared to determine the extent to which JBI correctly recognized Bergdahl.

### 2.2 Thematic content analysis

We used simple inductive content analysis to determine the extent to which the content of rebuttals matched the content of their respective critiques. JBI was examined to identify points attributed to Bergdahl (e.g., Bergdahl claims that….). Thereafter, Bergdahl was examined to identify passages that matched the attributions made by JBI. These passages were coded, and semantic relationships were established between the coded passages in Bergdahl and the matching primary rebutting claims made by JBI (e.g., rebutting statement *criticizes* critical statement). The passages in Bergdahl were then substantively contrasted with JBI's claims to determine the extent to which JBI's attributions captured the nature and extent of Bergdahl's statements. A rebuttal was determined to match a critique when all analytically relevant aspects of the rebuttal could be matched to passages in Bergdahl.

### 2.3 Argumentation analysis

Toulmin's approach to argumentation analysis, as discussed by Liakopoulos (2000), was used to both identify the primary claims JBI made and how these claims were supported. Liakopoulos’ approach was used as it is simple, well described, uncontroversial, and adequate for our purposes. This method involved identification and coding of passages of text in the paper by JBI as claims, supporting arguments, and backing for each supporting argument. Semantic relationships between the claims, arguments, and backing were then visually represented by creating networks in Atlas.ti to demonstrate the support JBI provided for each claim in their rebuttal. The backing for each claim of the rebuttal was then classified, using the startlingly clear guide “How to Disagree” by Graham (2008) into the categories: name‐calling, ad‐hominem, response to tone, contradiction, counterargument, refutation, or refutation of the central point. Rebuttals were determined to be adequately supported when all supporting arguments had recognizable backing. Refutation was taken as the minimum standard given the provision of appropriate and adequate backing. Appropriate and adequate backing, mirroring that required to justify models or methods in reports of qualitative research, required sound logical argument or appropriate citation.

## 3 RESULTS

This essay tested an application of the standard integrity to a case: JBI's response to Bergdahl. We, therefore, have two levels of result: substantive and methodological. These are discussed in this order as the substantive results are relevant in presenting our methodological results.

### 3.1 Substantive results^1^

To what extent does JBI recognize bergdahl's definitions of key terms?

Sound argument requires participants to properly recognize the claims each are making. This capacity, as noted above, is consistent with JBI's expectation that reviewers be able correctly to understand the texts they examine. In the exchange between Bergdahl and JBI the terms meta‐aggregation, meta‐synthesis, and the qualitative sciences figure prominently so are used to test JBI's recognition of Bergdahl's definitions of those terms.

JBI is consistent with Bergdahl in recognizing that meta‐aggregation is data‐grounded/inductive; based on interpretation; and aims to generate a common objective meaning. JBI misrecognizes Bergdahl, however, on every point that is relevant to practice. Where Bergdahl presents meta‐aggregation as reducing findings; sorting away explanations; stripping meaning; and of no use to practice, JBI works with a meta‐aggregation that synthesizes findings; generates explanatory statements; inclusively recognizes the meaning of findings; and provides useful guidance to practice.

JBI is consistent in recognizing meta‐synthesis as a synthesis of qualitative findings. However, as JBI do not provide a substantive discussion of their understanding of meta‐synthesis, it is not possible to test their agreement with the detailed description proposed by Bergdahl.

JBI is consistent with Bergdahl in their recognition that the qualitative sciences aim to: (a) clarify meaning and (b) serve understanding of findings. JBI, however, silently rejects several aspects of Bergdahl's definition. JBI, for instance, proposes that qualitative sciences: (a) facilitate *general* conclusions to serve understanding of findings (emphasis “general”) and (b) *consider* findings, which is not consistent with Bergdahl who sees them to (emphasis “consider”): (a) create *specific* theory within *context* to serve interpretation and understanding of findings (emphasis “specific”) and (b) *construct* and *critically question* instead of merely consider findings (emphasis “construct” “critically question”).

Where JBI provides sufficient data, it appears that JBI does not adequately recognize key claims made by Bergdahl.

To what extent does the content of JBI's rebuttals match the content of the matching critiques?

To what extent do JBI's rebuttals reference recognizable backing?

One of the capacities required to test the congruence of a qualitative study is the ability to determine which components are related, how they are related; and how well they fit each other. The parallel in argumentation is that rebuttals must relate and respond to their intended critique. Taking the next step, the selection of each of the components used in a qualitative study must be acceptably justified. As is the case in review of studies, in this essay both logic and evidence provide acceptable backing.

JBI contends that it “…is unclear why Bergdahl has chosen to contrast meta‐aggregation solely with meta‐ethnography” (p. 2) and backs this argument with the assertion that the “critique provided by Bergdahl is overshadowed by a lack of clarity on the terms used across the qualitative synthesis community” (p. 3). Bergdahl, however, did not solely contrast meta‐aggregation with meta‐ethnography and she argued that researchers “must also take a critical stance towards the methods used” (p. 5). In this case, JBI has neither correctly recognized the relevant components of Bergdahl's critique nor provided recognizable backing.

JBI contends that “The claim that the endpoint of a qualitative evidence synthesis ends with a theory positions Bergdahl with the general belief that only one philosophical paradigm is appropriate for all types of qualitative syntheses” (p. 2). JBI backs this claim with the assertion that “Methodological diversity should be encouraged to acknowledge and meet the broad range of knowledge needs associated with scholarship and theory generation” (p. 2) and backs this with the assertion that “this perspective is aligned with pragmatism” (p. 3).

Bergdahl does claim that an aim of the qualitative review is the theory which is justified by the assertion, never countered by JBI, that theory is a necessary condition for the interpretation of results. In this response, JBI binds advocacy for theory creation to the belief that only one philosophical paradigm is appropriate for all types of qualitative synthesis, and then falsely attributes this position to Bergdahl. At no point does JBI: (a) identify the paradigm trapping Bergdahl; (b) explain how advocacy for theory generation necessarily traps scholars within a single paradigm; or (c) help the reader understand how JBI's immediate endorsement of pragmatism is anything other than entrapment within a single paradigm. Furthermore, the “alignment” deployed to back the legitimacy of JBI's aggregation requires that the target to which their practice is aligned—pragmatism—is itself coherent, compatible with the goals of review, and sympathetic to atheoretical aggregation across heterogeneous studies. The same source cited by JBI to back endorsement of pragmatism, Cherryholmes (1992), states that “there are many versions of pragmatism” (p. 13). This would suggest that there may be no coherent referent to support alignment. Cherryholmes goes on to assert that the revolutionary contribution of pragmatism, which distinguishes it from scientific realism, is a commitment to providing the reader with a diversity of mutually distinct means. These means serve to reach their stated objective (e.g., improving literacy) that may then be assessed according to values, esthetics, politics; and social and normative preferences (e.g., a desirable community). In this connection, Cherryholmes attributes to Dewey the statement "Not everything that works is desirable, not every belief that is 'true' is to be acted upon" (p. 14). The declared interest of this pragmatism is to afford policy makers with a diversity of effective options that vary in their coincident effects. It is not clear in JBI's text how retention of the detail required to predict coincident effects is compatible with the decontextualizing aggregation they practice, nor is it clear that the purpose of JBI review is to identify mutually distinct equally effective means from which practitioners may choose based on preferred coincident effects.

JBI has thus incorrectly recognized and invented portions of Bergdahl's critique, as well as provided backing that either fails to rise to the level of refutation, or merely contradicts the point they are attempting to make.

With respect to meta‐synthesis, JBI states that they “…agree that the transfer of these presuppositions to methodology for the health professions is worthwhile. However, we are concerned with the notion that it is the only perspective of value for the health care sector” (p. 2). Bergdahl does not claim that meta‐synthesis is the only valuable perspective for the health‐care sector. She states that “meta‐synthesis could serve to re‐interpret, compare and translate different qualitative studies, using a different conceptual apparatus, into a consolidated knowledge of fundamental importance to nursing care practitioners” (p. 7). Bergdahl goes on to state that all results from meta‐research always require epistemological discussion—especially within the health‐care sector—for the results to acquire value. JBI misreads Bergdahl (“Meta‐synthesis could serve…” [[emphasis “could”]) and JBI does not address Bergdahl's claim that meta‐research always requires epistemological consideration. Taking the next step, none of the arguments provided by JBI are accompanied by recognizable backing. For example, to back the argument that sensitivity to theory and methods is not required, JBI states that the standards suggested by Bergdahl, sensitivity to theory and methods by the reviewer, are not appropriate:

Theory building as the primary pursuit of knowledge synthesis requires caution when extrapolating to practising health professions whose primary interests are in the delivery of care … expert clinical practice is founded on theory and evidenced through ‘thinking in action.' (p. 2)

The claim that the primary purpose meta‐synthesis is theory building, which JBI attributes to Bergdahl, cannot be found in her intervention, and the assertion that thinking in action is what counts as sound evidence for clinical practice is not accompanied by a description or backing. JBI has thus incorrectly recognized and invented portions of Bergdahl's critique. They then fail to provide sound argument or recognizable backing to support their response.

JBI states that the “adoption of a pragmatist perspective for meta‐aggregation is compatible with the epistemological, philosophical, and ethical traditions of qualitative research and is therefore justified as a proper scientific method” (p. 2). This assertion is presented as a rebuttal of Bergdahl's argument that “aggregation seems incompatible with the qualitative tradition” (p. 2), which is backed by the cited assertions both that “leading voices in the interpretivist qualitative research tradition are explicitly against generalization” (p. 4) and that JBIs approach to meta‐aggregation “stands in contradiction to what is understood as good, post‐positivistic, scientific practice” (p. 4) since “interpretive methods reject the notion of an objective truth that can be found, and verified, by empirical methods” (p. 4). Furthermore, Bergdahl does not claim that meta‐aggregation is not a proper scientific method. Her claim is that it is “a different approach that is in contrast to meta‐synthesis and should not be considered a form of meta‐synthesis. If that is correct, meta‐aggregation cannot be justified as a proper scientific practice in terms of being a form of meta‐synthesis” (p. 1).

JBI does not correctly read Bergdahl where she states that “meta‐aggregation seems incompatible with the qualitative research traditions” (emphasis “seems”) and that “meta‐aggregation is not a proper scientific method in terms of being a form of meta‐synthesis” (emphasis “in terms of being a form of meta‐synthesis") and JBI fails to address the supporting discussion of postpositivist science. Even granting that JBI's reading was adequate, they would not have refuted Bergdahl. JBI does not explain why adoption of a pragmatist perspective for meta‐aggregation is compatible with the qualitative research tradition as described by JBI, with reference to postpositivism and construction, so their response can only be recognized as a counter. JBI has not correctly understood or fully addressed Bergdahl, and the responses made by JBI are not adequately backed.

With reference to a study mentioned by Bergdahl, Lamb et al. (2008) (hereafter Lamb) as exemplifying how meta‐aggregation “moves knowledge backward, from specific and actionable knowledge to broad generalization” (Bergdahl, p. 5), JBI states “We credit Bergdahl for the comment that the example review cited from Lamb and colleagues would have had different results if it were conducted from an interpretive perspective. We doubt, however, whether it should have had different results” (p. 2). Bergdahl does not claim that the example review of Lamb would or should have had different results if it were conducted from an interpretative perspective (emphasis “would,” “should”). Instead, Bergdahl claims that the example review of Lamb could have had different results if it were conducted from an interpretive, thus meta‐synthesis perspective (emphasis “could”). Using logic, Bergdahl suggests that meta‐synthesis could produce something more than generalization because it is sensitive to theory and context. This supports Bergdahl's argument that meta‐synthesis could “serve to re‐interpret, compare and translate different qualitative studies, using a different conceptual apparatus, into a consolidated knowledge of fundamental importance to nursing care practitioners” (p. 7) (emphasis “could”). JBI misreads the claims made by Bergdahl concerning the review paper of Lamb they fail to see the difference between could and should and they fail to address the arguments Bergdahl uses to support her claims. Even if JBI correctly read Bergdahl on Lamb, their response would only amount to a contradiction.

JBI asserts that in their approach to meta‐aggregation “findings are grouped based on similarity of meaning (as Bergdahl rightly highlights) into categories, from which synthesised findings are constructed. Bergdahl takes issue with this concept of ‘similarity in meaning’; however, that phrase does not just mean simply matching like with like” (p. 3). JBI is mistaken in their interpretation of Bergdahl's “similarity in meaning.” Bergdahl does not say that “similarity in meaning” in meta‐aggregation is the matching of like with like. She likens the JBI approach to “generating findings using the highest common denominator in mathematics: the more cases one adds, the fewer common factors there are going to be. Thus, very few and common abstract attributes are left, and any context‐dependent attribute or property is sorted away” (p. 4). Bergdahl does claim this search for “similarity in meaning” in meta‐aggregation to be a weakness of the method as interpretation “towards common meaning will naturally evolve from specific terms to less specific and more general, less informative and abstract terms” (p. 4), which brings the risk that the searching for common meaning advocated by JBI results in “neglecting, crucial contradictory findings in specific contexts.” As such “results run the risk of becoming meaningless abstractions that do not say anything profound about how things work” (p. 4).

JBI has not correctly read the claims Bergdahl, addressed all important supporting arguments or provided adequate backing for their own claims.

### 3.2 Methodological results

While our substantive results demonstrate that: those who conduct review of qualitative research may declare standards that are relevant for consideration of their contributions to debates over methods; and that these standards allow to produce results that are possible to interpret and defend. We did encounter several challenges.

In our analysis, we started from JBI's rebuttals and, then selected for each rebuttal from Bergdahl only those data that were immediately relevant. There were several dimensions to Bergdahl's critique that JBI did not mention. This limited the claims we could make to those individual rebuttals and not to the extent to which JBI answers the entire critique.

In our examination of both Bergdahl and JBI, we found that components of claims were not consistently clustered. Identifying all the relevant components of a claim required careful repetitive reading of the entire text. A side‐effect of the scattering of components was that the relationships between components were not clear at times. While we did resolve this ambiguity, the path we chose, of making very charitable assumptions, biases our results.

We found that JBI's rebuttals at times did not structurally match Bergdahl's critiques. This meant, for instance, that while Bergdahl provided evidence or logic to back all the arguments that supported a particular critique, JBI's response at times missed branches, added branches, or operated at a different depth. The lack of structural identity between the critique and response introduced substantial missing data. While the third research question, which queried the presence and nature of backing, was sensitive to depth (i.e., are claims backed?), it was not sensitive to missing branches (i.e., Are all component claims in Bergdahl addressed?) so, our method does not provide comprehensive coverage of data.

Finally, our analysis focuses entirely on the behavior of JBI. We were constrained to the examination of JBI, as we could not justify using JBI's standards to assess Bergdahl and as we could not find an equivalent set of standards that covered Bergdahl's behavior. In this case, where we found standards to cover only one party's practice and where the practice of that party was found to be severely wanting, and despite several methodological choices that bias results in JBI's favor, our efforts may appear biased against JBI.

## 4 DISCUSSION

Our essay started from the premise that those who take part in debates over methods ought to speak with integrity. To that end, we examined JBI's declared review guidelines for standards that might be relevant in assessing their contributions to an academic debate. We found that the review protocol created by JBI places great importance on congruence and that it is possible to translate these guidelines to cover their participation in discussions of their method. We expected JBI to (1) correctly interpret statements of others; (2) to comprehensively recognize arguments; and (3) to provide responses that were supported by logic or evidence. The text written by Bergdahl, which clearly references well‐established arguments, appeared to pose a direct threat to the foundations on which the work done by JBI rests. Therefore, we expected JBI to take Bergdahl seriously.

The first capacity JBI was tested for was their ability to understand the texts they examine. Our standard for testing this understanding was performative: the extent to which JBI correctly recognizes, either through adoption or justified contestation, the definitions given by Bergdahl to key terms. The domain analysis we undertook to examine this, found serious unjustified inconsistencies between Bergdahl and JBI. Furthermore, our subsequent argument analysis frequently tripped over instances where JBI, without comment, used their own definitions rather than Bergdahl's. Since JBI failed to transparently account for Bergdahl's use and definition of key terms, they did not demonstrate that they are able to understand a text.

The second capacity JBI was tested for was their ability to correctly recognize all relevant components of an argument. Our standard for testing this recognition was, again, performative: the extent to which JBI's responses address the arguments used by Bergdahl to support her critical claims. We found many instances where JBI failed to respond to key arguments supporting the critical claims made by Bergdahl. JBI did not demonstrate that they are able to recognize all analytically relevant arguments in a text.

The third capacity JBI was tested for was JBI's understanding of the standards of evidence required to support claims within qualitative research. Our expectation here was that JBI would provide adequate and appropriate backing for each argument made in countering the critiques made by Bergdahl. We found that JBI failed to provide any backing for assertions in most cases and that, in one case that appears to be fundamental to the legitimacy of their project, the backing offered seemed to misrepresent the source referenced. JBI has thus failed to demonstrate either structural (i.e., important claims require backing) or substantive (i.e., backing must be adequate) respect for the standards of evidence within qualitative research that they, themselves, endorse.

## 5 CONCLUSION

This study was motivated by interest in finding means to assess arguments over methods within the low consensus field of qualitative review. To that end, we decided to test integrity: the extent to which declared standards for review predict practice in its defence. We used JBI's rebuttal of Bergdahl's critique as an example. We found that JBI declares a strong interest in congruence in their own protocols for review, and that this standard is relevant for assessing their rebuttal of a critique. On application, we found that JBI did not correctly address or recognize Bergdahl's definitions of the key terms “meta‐aggregation,” “meta‐synthesis,” and “qualitative sciences”; that the rebuttals JBI provided as response to Bergdahl's critique did not match their content; and that their rebuttals often lacked adequate backing, thereby failing to refute any of Bergdahl's critiques. We thus found that JBI's rebuttal of Bergdahl lacked integrity.

Turning to our methodological interest in integrity, if the case we selected is any indicator, it seems that integrity is a workable standard to adopt when examining debates over review methods when parties disagree on standards. However, use of integrity is both structurally and substantively vulnerable.

## CONFLICT OF INTERESTS

The authors declare that there are no conflict of interests.

## Supporting information

Supporting information.

## Data Availability

The data are copyright journal articles so are not possible to submit to a public respository. The data set is available on request.

## Footnotes

^1^
Full substantive results are appended as supplementary Information.
