# Supplementary material for: The standard of integrity may be useful when assessing arguments over qualitative review methods: The case of the Joanna Briggs Institute's rebuttal of a fundamental critique
Source: Nurs Inq. 2021 Sep 25;29(3):e12465. doi: 10.1111/nin.12465 (PMC9539601; doi:10.1111/nin.12465)

**Supplementary Information: Full Results**

Does JBI correctly recognize Bergdahl?

Meta-aggregation

According to JBI, meta-aggregation is one the more appropriately considered forms of qualitative evidence synthesis, which entails the data-grounded interpretative grouping of two or more findings that address a similar phenomenon or have a similar meaning, into meaning-based categories. These categories are synthesized into final explanatory statements which comprise a summary of the best available evidence from data and reflect the review team's perspective on what the combined inclusive meaning of the findings represents. According to JBI, this provides useful guidance to clinicians and policy‐makers, and helps to inform practice and clinical decision making.

According to Bergdahl, meta-aggregation is the inductive generalization of research findings through the performance of phenomenological reduction and interpretation where context‐dependent attributes, properties, models and theories that provide contextual explanations and describe the relationship between phenomena, (both similarities and differences) are sorted away, to generate a common objective meaning of the findings. According to Bergdahl, the result of meta-aggregation therefore comprises abstractions and generalizations that do not offer a greater understanding of phenomena, but that are devoid of meaning and of no use to intervention and implementation.

JBI is consistent with Bergdahl in recognizing that meta-aggregation is: a) data-grounded/inductive; b) based on interpretation, and; c) aiming to generate a common objective meaning. They however fail to entirely recognize Bergdahl in her definition of meta-aggregation, as as JBI deem meta-aggregation to: a) entail a synthesis of findings; b) generate explanatory statements; c) comprise the inclusive meaning of findings, and; d) provide a useful guidance to practice, whereas Bergdahl deems it to: a) entail a reduction of findings; b) sort away explanations; c) devoid meaning of findings, and d) be of no use to practice.

Meta-synthesis

According to JBI, meta-synthesis, or - as they like to refer to it - qualitative evidence synthesis, entails the pooling or synthesis of qualitative systematic reviews and findings.

According to Bergdahl, meta‐synthesis is the last phase in a meta-study that entails the interpretive and creative integration of both similarities and differences between existing qualitative findings - which themselves are interpretive syntheses of data (including the phenomenologies, ethnographies, grounded theories, and other coherent descriptions or explanations of phenomena, events, or cases that are the hallmark findings of qualitative research) - using a different conceptual apparatus than the ones used in the individual studies the findings originate from. According to Bergdahl, meta-synthesis thereby offers a theoretical integration of the research findings: a new, integrated, more complete, and clear interpretation and representation of the original findings to make the complexity of their social interaction more understandable. According to Bergdahl, this resulting theoretical integration could possibly: a) serve as a starting point for theory development, and; b) contribute to scientific knowledge of fundamental importance to inter alia nursing care practitioners.

JBI and Bergdahl concur in their recognition of meta-synthesis as a synthesis of qualitative findings. However, as JBI do not provide a more discussion of their understanding of meta-synthesis, it is not possible to test their agreement with the definition proposed by Bergdahl (2019).

Qualitative sciences

According to JBI, the qualitative sciences rest on the pragmatist perspective, in which research seeks to clarify meaning and considers the consequences of actions, based on the aggregation of well-specified data that assist with auditability of the reviewers’ decisions in understanding the findings, and facilitate general conclusions.

According to Bergdahl, the qualitative sciences rest on the constructivist/interpretivist perspective, where: a) the positivist notion that underlies quantitative sciences - that objective meaning exists - is rejected; b) the core comprises critical thinking, and; c) the aim and hallmark entail the generation of specific theory to serve the interpretation of statements and their meaning within context. According to Bergdahl, all knowledge in the social sciences is unique, context‐bound, and constructed through trial and error - creative interpretations and critical questioning and evaluation of interpretations, hypotheses and theory - instead of through phenomenological statistical methods.

JBI and Bergdahl concur in their recognitions that the qualitative sciences aim to: a) clarify meaning, and; b) serve understanding of findings.JBI however fails to correctly recognize Bergdahl's definition of social/qualitative sciences asJBI for instance proposes that qualitative sciences: a) facilitate *general* conclusions to serve understanding of findings, and; b) *consider* findings, whereas Bergdahl sees them to: a) create *specific* theory within *context* to serve interpretation and understanding of findings, and; b) *construct* and *critically question* instead of merely consider findings.

Are JBI’s responses adequate

JBI responds to five critiques that they attribute to Bergdahl. This section reports on both the adequacy of JBI’s understanding of Bergdahl, as well as the nature and adequacy of the support given each response.

Claim1: *It is unclear why Bergdahl has chosen to contrast meta‐aggregation solely with meta‐ethnography rather than considering its place within qualitative evidence synthesis more broadly. In the light of the uncertainty around language, using these terms to situate specific beliefs about scientific credibility could be considered a false flag.*- JBI

Bergdahl did not solely contrast meta-aggregation with meta-ethnography:

*While the result of aggregation is a generalized statement, the result of a meta‐synthesis is a theoretical integration of the research findings ... This integration—synthesis—is ... exactly the opposite of reduction and finding a common meaning … Prescriptions … or theory … derived from meta‐study research cannot be regarded as the only possible findings that could be drawn from the body of available research … In contrast, meta‐aggregation …” - Bergdahl*

Bergdahl does not claim that meta-synthesis, meta-aggregation and meta-ethnography situate specific beliefs about the credibility:

*A researcher must also take a critical stance towards the methods used. The researchers must explain how the method can contribute to knowledge development … Simply because a study follows a described methodology does not, of course, mean that all findings in that study are valid - Bergdahl*

JBI back their claim by stating:

*The critique provided by Bergdahl is overshadowed by a lack of clarity on the terms used across the qualitative synthesis community … Meta‐synthesis is a contested term with multiple meanings … Meta‐aggregation is one of many different methodologies that are more appropriately considered forms of ‘qualitative evidence synthesis’. - JBI*

Though JBI address why one might refrain from using the term meta-synthesis to situate specific beliefs about scientific credibility (the lack of clarity in language), they do not specify why one should also refrain from using the other terms they mention, meta-aggregation and meta-ethnography, in situating scientific credibility nor does JBI address why one should not contrast meta-aggregation solely with meta-ethnography but should consider its place in qualitative evidence synthesis more broadly. The statement used as argument - that meta-aggregation is one of the many different methodologies that is a more appropriately considered form of qualitative evidence synthesis - does not actually provide an explanation for why one should consider the place of meta‐aggregation within qualitative evidence synthesis more broadly. The response provided by JBI is, at best, a counter. The argument map for Claim 1 can be found in Figure 1.

JBI has neither correctly recognized the relevant components of Bergdahl’s critique nor provided recognizable backing.


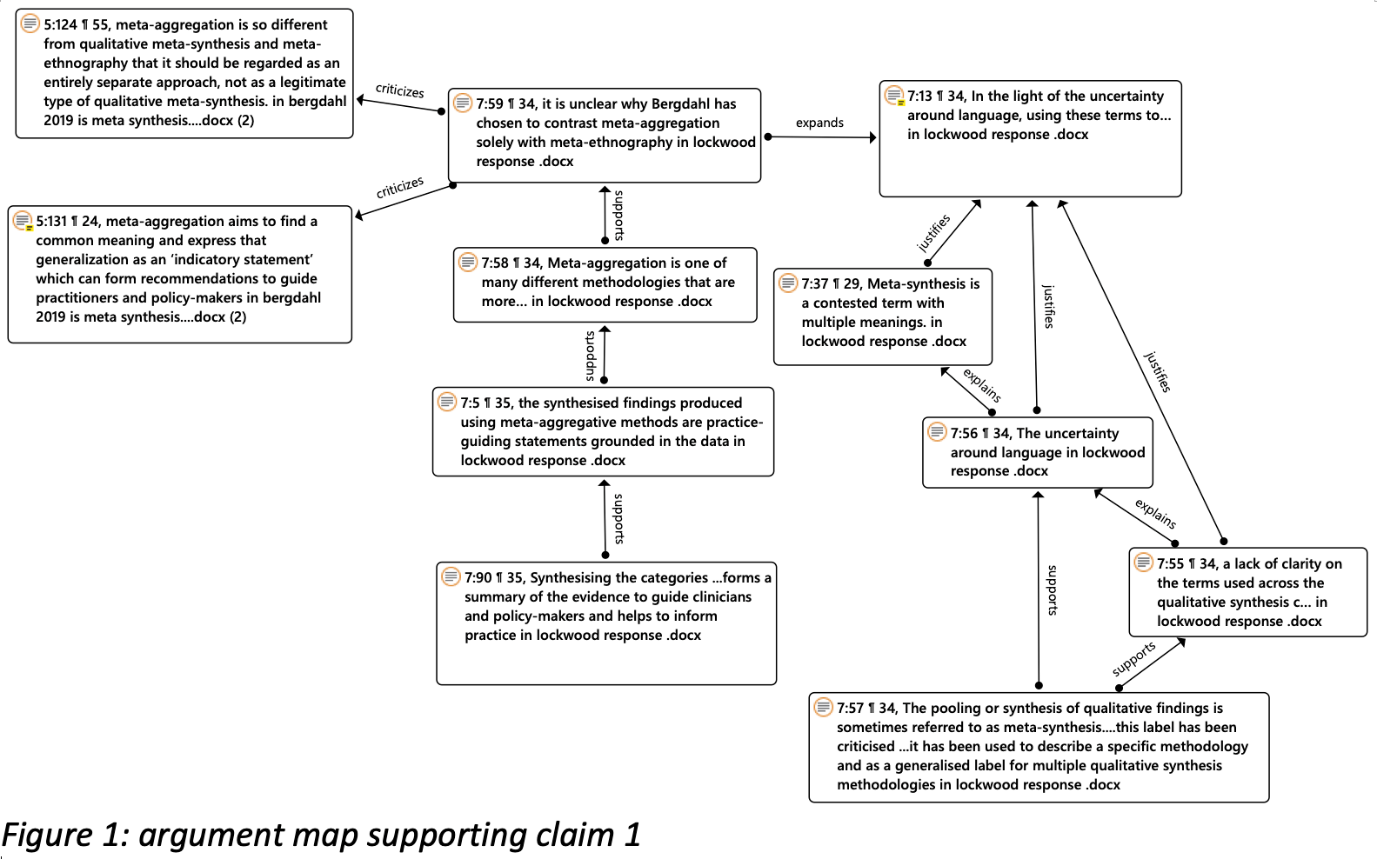


Claim 2: The claim that the endpoint of a qualitative evidence synthesis ends with a theory positions Bergdahl with the general belief that only one philosophical paradigm is appropriate for all types of qualitative syntheses. - JBI

Bergdahl does claim that theory is the aim of science that qualitative review serves:

*As I see it, theory is the aim of science, and there can be no science … without theory ... Meta‐synthesis would be a possible way to use existing research as a starting point for theory development … It is the synthesizing process that enables the meta‐researchers to clarify which types of phenomena exist and the causal relationships and intentional reasons behind different phenomena ... In that way, meta‐synthesis aims to create theory and thereby contribute to scientific knowledge*. - Bergdahl

Bergdahl furthermore claims that:

*Aggregation of disjointed ‘results’ cannot constitute scientific evidence, since there is no theoretical framework from which one can test or even interpret individual statements* - Bergdahl

JBI’s responds to Bergdahl by stating:

*We differ from Bergdahl in that we believe different philosophical stances are applicable to different situations and review contexts … Methodological diversity should be encouraged in order to acknowledge and meet the broad range of knowledge needs associated with scholarship and theory generation, as well as policy and practice … Meta‐aggregation works well … This perspective is aligned with pragmatism … offer a new understanding for clinicians and academics alike -* JBI

In this response, JBI binds advocacy for theory creation to the belief that only one philosophical paradigm is appropriate for all types of qualitative synthesis, and then falsely attributes this position to Bergdahl. At no point does JBI: a) identify the paradigm trapping Bergdahl; b) explain how advocacy for theory generation necessarily traps scholars within a single paradigm, or; c) help the reader understand how JBI’s immediate endorsement of pragmatism is anything other than entrapment within a single paradigm. JBI uses usefulness and alignment to counter Bergdahl’s claim that aggregation cannot constitute scientific evidence. JBI does not provide backing to support their tacit argument that “perceived usefulness is a reasonable substitute for scientific merit”, Furthermore, the ‘alignment’ deployed to back the legitimacy of JBIs aggregation requires that the target to which their practice is aligned, pragmatism, is itself coherent, compatible with the goals of review, and sympathetic to atheoretical aggregation across heterogeneous studies. The same source cited by JBI to back endorsement of pragmatism, Cherryholmes (1992), states that “there are many versions of pragmatism” (p. 13), which would suggest that there may be no coherent referent to support alignment. Cherryholmes goes on to assert that the revolutionary contribution of pragmatism, which distinguishes it from scientific realism, is a commitment to providing the reader with a diversity of mutually distinct means by which they may reach their stated objective (e.g. improving literacy) that may then be assessed according to values, aesthetics, politics and social and normative preferences (e.g. a desirable community). In this connection, Cherryholmes attributes to Dewey the statement ‘Not everything that works is desirable, not every belief that is "true" is to be acted upon’ (p. 14). The declared interest of the pragmatism described in the source cited by JBI serves to afford a diversity of effective options that vary in their coincident effects. It is not clear in JBI’s text how retention of the detail required to predict coincident effects is compatible with the forms of aggregation they practice. Furthermore, it is not clear that the purpose of JBI review is to identify mutually distinct effective means from which practitioners may choose based on preferred coincident effects. The argument map for Claim 2 can be found in Figure 2.

JBI has thus incorrectly recognized and invented portions of Bergdahl’s critique, as well as provided backing that either fails to rise to the level of refutation, or merely contradicts the point they are attempting to make.


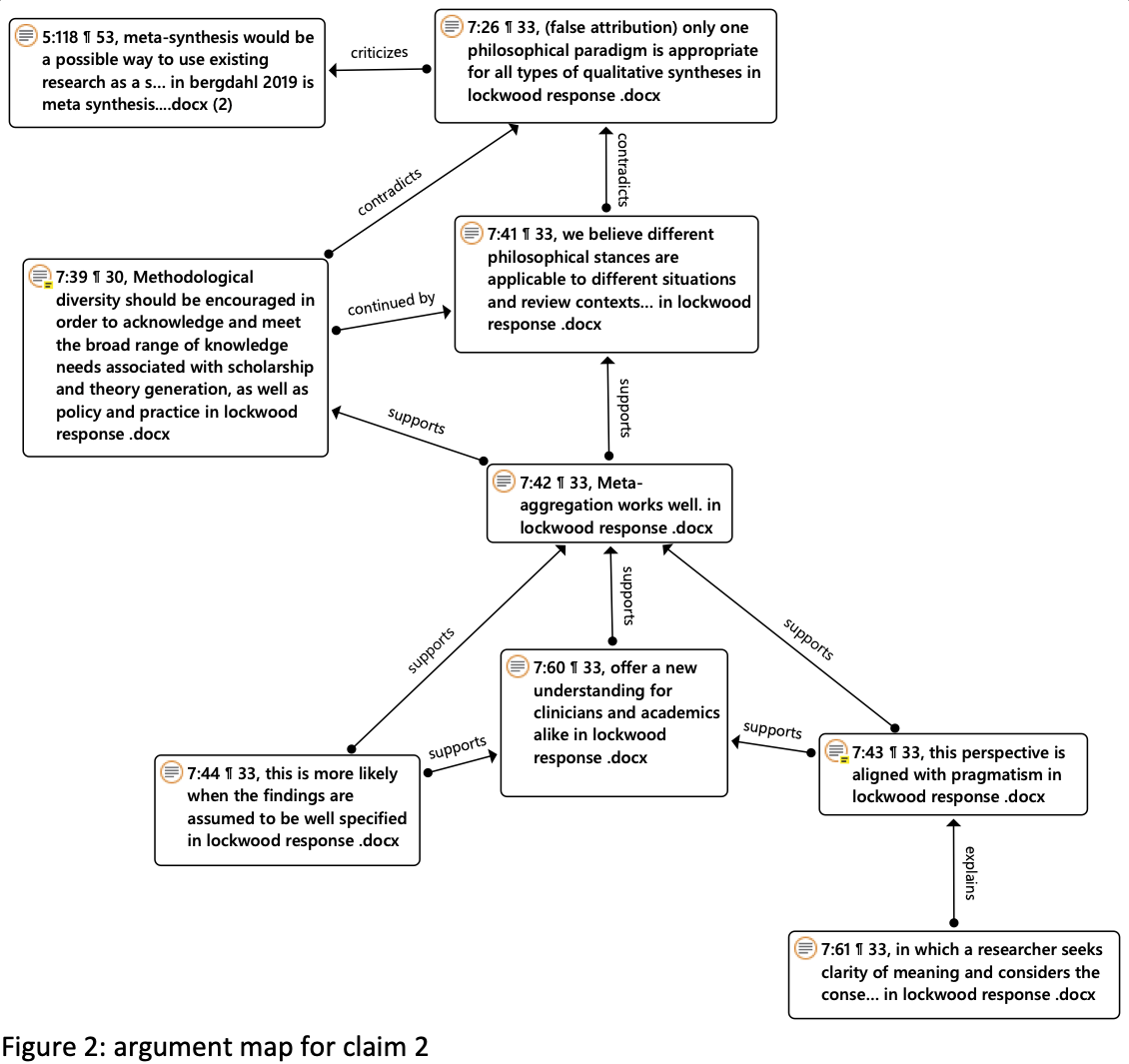


Claim 3: “We agree that the transfer of these presuppositions [of meta-synthesis within the social sciences] to methodology for the health professions is worthwhile. However, we are concerned with the notion that it is the only perspective of value for the health care sector. - JBI

Bergdahl does not claim that meta-synthesis is the only valuable perspective for the health care sector. She states, with emphasis added, that:

*Meta‐synthesis* ***could*** *serve to re‐interpret, compare and translate different qualitative studies, using a different conceptual apparatus, into a consolidated knowledge of fundamental importance to nursing care practitioners.*- Bergdahl (emphasis added)

She furthermore states that all results from meta-research always require epistemological discussion - especially within the healthcare sector - for the results to acquire value. That results should always be interpreted in light of the method and context they were found:

*We need to have a sound epistemological discussion, especially within the qualitative nursing research community … to debate the core characteristics that determine whether we can call a result scientific… Prescriptions … derived from meta‐study research cannot be regarded as the only possible findings that could be drawn from the body of available research, but rather as those findings constructed by a specific meta‐synthesis at a given point in accordance to their own interpretative skills.* - Bergdahl

JBI misreads Bergdahl (“*Meta‐synthesis could serve…”*) and JBI does not address Bergdahl’s claim that meta-research always requires consideration of context and methods. Taking the next step, none of the arguments provided by JBI are accompanied by recognizable backing. For example, to back the argument that sensitivity to theory and methods is not required, JBI states that the standards suggested by Bergdahl, sensitivity to theory and methods by the reviewer, are not appropriate:

*Theory building as the primary pursuit of knowledge synthesis requires caution when extrapolating to practising health professions whose primary interests are in the delivery of care … expert clinical practice is founded on theory and evidenced through ‘thinking in action’. - JBI*

The argument put forward here, that what counts as sound evidence for clinical practice is different, is not accompanied by backing that defends the asserted difference between clinical and scientific evidence. It is not clear how findings that have been stripped of theory well support expert clinical practice that is founded on theory and the ‘thinking in action’ invoked here, is not detectably linked to any other part of their argument. The argument map for Claim 3 can be found in Figure 3.

JBI has thus incorrectly recognized and invented portions of Bergdahl’s critique. They then fail to provide sound argument or recognizable backing to support their response.


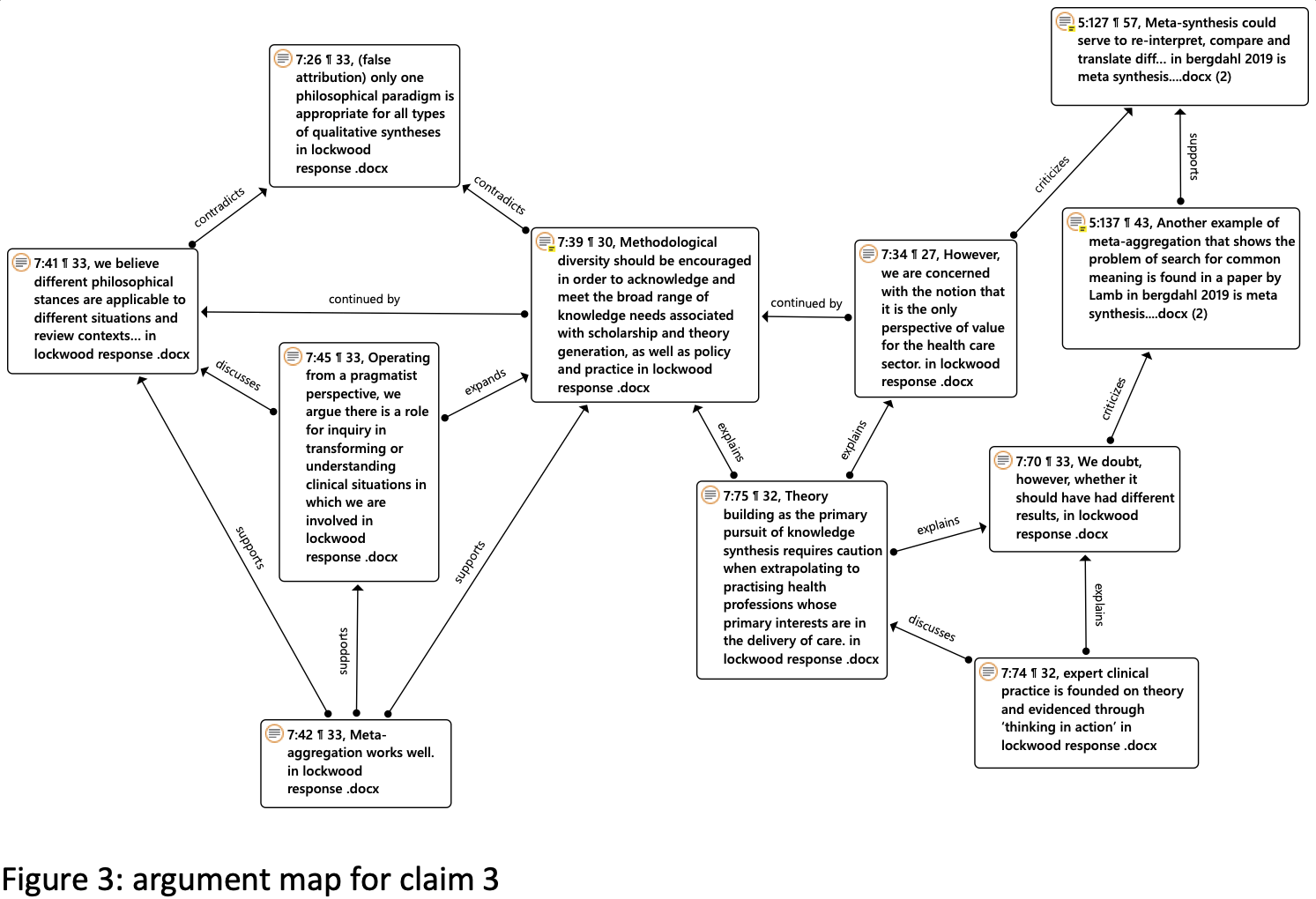


Claim 4: “We respectfully disagree with the central tenets posed by Bergdahl, and we would like to advance the following points: … The adoption of a pragmatist perspective for meta‐aggregation is compatible with the epistemological, philosophical and ethical traditions of qualitative research and is therefore justified as a proper scientific method.” - JBI

Bergdahl places the claim that aggregation is incompatible with the qualitative research tradition in a careful context. She states:

*Aggregation seems incompatible with the qualitative tradition … Meta‐aggregation focuses on common meaning and generalization to a common meaning. Leading voices in the interpretivist qualitative research tradition are explicitly against generalization … Finding a common meaning among several underlying findings and categories … stands in contradiction to what is understood as good, post‐positivistic, scientific practice … qualitative science is incompatible with positivism, since constructivist and interpretive methods reject the notion of an objective truth that can be found, and verified, by empirical methods.* - Bergdahl

Furthermore, Bergdahl is also not claiming that meta-aggregation is not a proper scientific method in general, but merely in terms of being a form of meta-synthesis.

*I argue that meta‐aggregation is a different approach that is in contrast to meta‐synthesis and should not be considered a form of meta‐synthesis. If that is correct, meta‐aggregation cannot be justified as a proper scientific practice in terms of being a form of meta‐synthesis*. - Bergdahl

JBI does not correctly read Bergdahl where she states that “*meta-aggregation seems incompatible with the qualitative research traditions*” and that “*meta-aggregation is not a proper scientific method in terms of being a form of meta-synthesis*” and JBI fails entirely to address the supporting discussion of post-positivist science. The argument map for Claim 4 can be found in Figure 4.

Even granting that JBI’s reading was adequate, they would not have refuted Bergdahl. JBI does not explain why adoption of a pragmatist perspective for meta‐aggregation is compatible with the qualitative research tradition as described by JBI, with reference to post-positivism and construction, so their response can only be recognized as a counter.

JBI has not correctly understood or fully addressed Bergdahl, and the responses made by JBI are not adequately backed. Therefore, their response can merely be recognized as a misguided and partial counter.


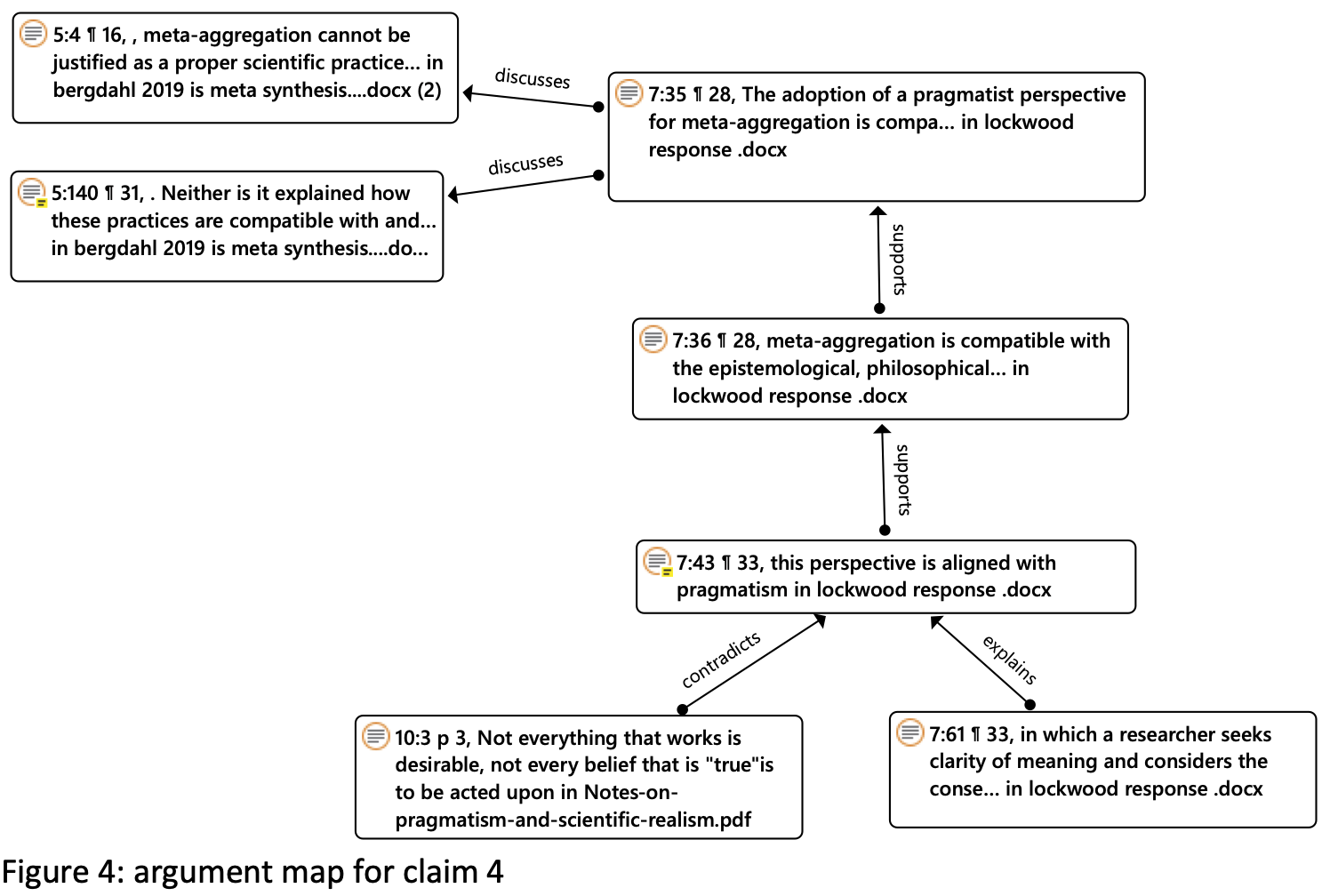


Claim 5: “We credit Bergdahl (2018) for the comment that the example review cited from Lamb and colleagues would have had different results if it were conducted from an interpretive perspective. We doubt, however, whether it should have had different results.” - JBI

Bergdahl does not claim that the example review of Lamb et al. would or should have had different results if it were conducted from an interpretative perspective. Instead, Bergdahl claims that the example review of Lamb et al. (2008) could have had different results if it were conducted from an interpretive, thus meta-synthesis perspective. She states that the paper by Lamb et al. (2008) shows what they deem the problem of using meta-aggregation, the search for common meaning: she deems the paper to show that the used meta-aggregation moved specific and actionable knowledge to broad generalization.

*Another example of meta‐aggregation that shows the problem of search for common meaning is found in a paper by Lamb, Buchanan, Godfrey, Harrison, and Oakley (2008). These authors used meta‐aggregation to ‘…appraise and synthesize best available evidence on the psychosocial spiritual experience of elderly individuals recovering from stroke’ … This aggregation moves knowledge backward, from specific and actionable knowledge to broad generalization.* - Bergdahl

Bergdahl deems meta-synthesis to be able to determine something more than a generalization due to its comparative nature, such as a theoretical integration of context or patient group-specific findings. She therefore claims that meta-synthesis could serve as a way to re-interpret findings from qualitative studies into scientific knowledge for health care practice.

*However, we perform research to determine something more—how a stroke is life‐changing, how can we help specific groups, what is the cause of certain reactions in specific contexts, and so on ... Almost all the included studies have some context or patient group‐specific findings which get generalized away in the meta‐aggregation … while the result of aggregation is a generalized statement, the result of a meta‐synthesis is a theoretical integration of the research findings … Meta‐synthesis could serve to re‐interpret, compare and translate different qualitative studies, using a different conceptual apparatus, into a consolidated knowledge of fundamental importance to nursing care practitioners.* - Bergdahl

JBI misreads the claims made by Bergdahl concerning the review paper of Lamb et. al. (2008). JBI fail to see the difference between could and should and JBI fails to address the arguments Bergdahl uses to support her claims.

Even if JBI correctly read Bergdahl on Lamb, their response would only amount to a contradiction. This is the case as JBI fails to both explain why they think that the review of Lamb et al. (2008) should not have had different results, and provide any backing for this position. JBI have not: a) correctly read Bergdahl; b) addressed all important aspects of her argument, and; c) provided recognizable substantiation for their own claims. Therefore, their response is only recognizable as a misguided partial counter.

Claim 6: In the JBI approach to meta‐aggregation, findings are grouped based on similarity of meaning (as Bergdahl rightly highlights) into categories, from which synthesised findings are constructed. Bergdahl takes issue with this concept of ‘similarity in meaning’; however, that phrase does not just mean simply matching like with like.- JBI

JBI is mistaken in their interpretation of Bergdahl’s ‘similarity in meaning.’ Bergdahl does not say that ‘similarity in meaning’ in meta-aggregation is the matching of like with like. She describes similarity of meaning as follows:

*Finding a common meaning among several underlying findings and categories (Pearson et al., 2011; The Joanna Briggs Institute, 2014) could be likened to generating findings using the highest common denominator in mathematics: the more cases one adds, the fewer common factors there are going to be. Thus, very few and common abstract attributes are left, and any context‐dependent attribute or property is sorted away.* - Bergdahl

Bergdahl does claim this search for ‘similarity in meaning’ in meta-aggregation to be a weakness of the method:

*The core focus on common meaning in meta‐aggregation is a serious weakness in the approach described by Lockwood et al. (2015), Lockwood and Pearson (2013), Pearson et al. (2011) and in the JBI manual … Interpretation towards common meaning will naturally evolve from specific terms to less specific and more general, less informative and abstract terms … Adding new layers of abstraction pulls the aggregated result even further from its intended context and the original data. Searching for a common meaning also seems to run the risk of neglecting, crucial contradictory findings in specific contexts ... Findings that do not fit into that framework will be left out. Most importantly, there is no room for discussing contradictions and exceptions, nor method and theory. The results run the risk of becoming meaningless abstractions that do not say anything profound about how things work.* - Bergdahl

JBI’s reading of Bergdahl’s discussion of ‘similarity in meaning’ is not accurate insofar as Bergdahl’s “the combination of an increasing amount of findings until fewer common shared factors remain” is not the same as JBI’s “matching like with like”. Furthermore, JBI fails to address relevant supporting arguments, such as Bergdahl’s contention that a theory insensitive search for ‘similarity in meaning’ makes findings become meaningless abstractions by making specific terms more general, less informative and concrete due to it pulling findings from the intended context and original data, and neglecting the discussion or inclusion contradictory findings.

Even if JBI had correctly read Bergdahl, their response would not amount to a refutation. JBI merely counters Bergdahl by stating that in meta-aggregation, the concept entails the categorisation of a series of two or more like findings, which also reflect the review team's interpretative perspective on what the combined meaning of the findings represents.

*In meta‐aggregation, the operational definition of a category is a descriptive statement that meaningfully brings together key concepts arising from the aggregation of two or more like findings … accompanied by an explanatory statement that conveys the whole, inclusive meaning of a group of findings … the reviewer's interpretive process during categorisation must be linked explicitly and directly to the actual findings. Therefore, while categorisation is an interpretive process, it is both guided and constrained by the evidence extracted from the papers, rather than left to the review authors’ interpretive perspective*.- JBI

JBI do not provide an explanation for how it would be the case that interpretation would constitute more than merely matching multiple alike findings by a certain individual in a certain context. Interpretation might be deemed inherent to any process of matching findings by an individual in a context, due to which one might even come to consider that JBI do not state more than the claim they actually try to challenge Bergdahl on: that the concept ‘similarity in meaning’ in meta-aggregation is the matching of like with like. The argument map for Claim 6 can be found in Figure 5. JBI have thus not: a) correctly read the claims Bergdahl; b) addressed all important supporting arguments, and; c) provided a proper substantiation for their own claims. Their response is therefore, at best, a misguided and partial counter.


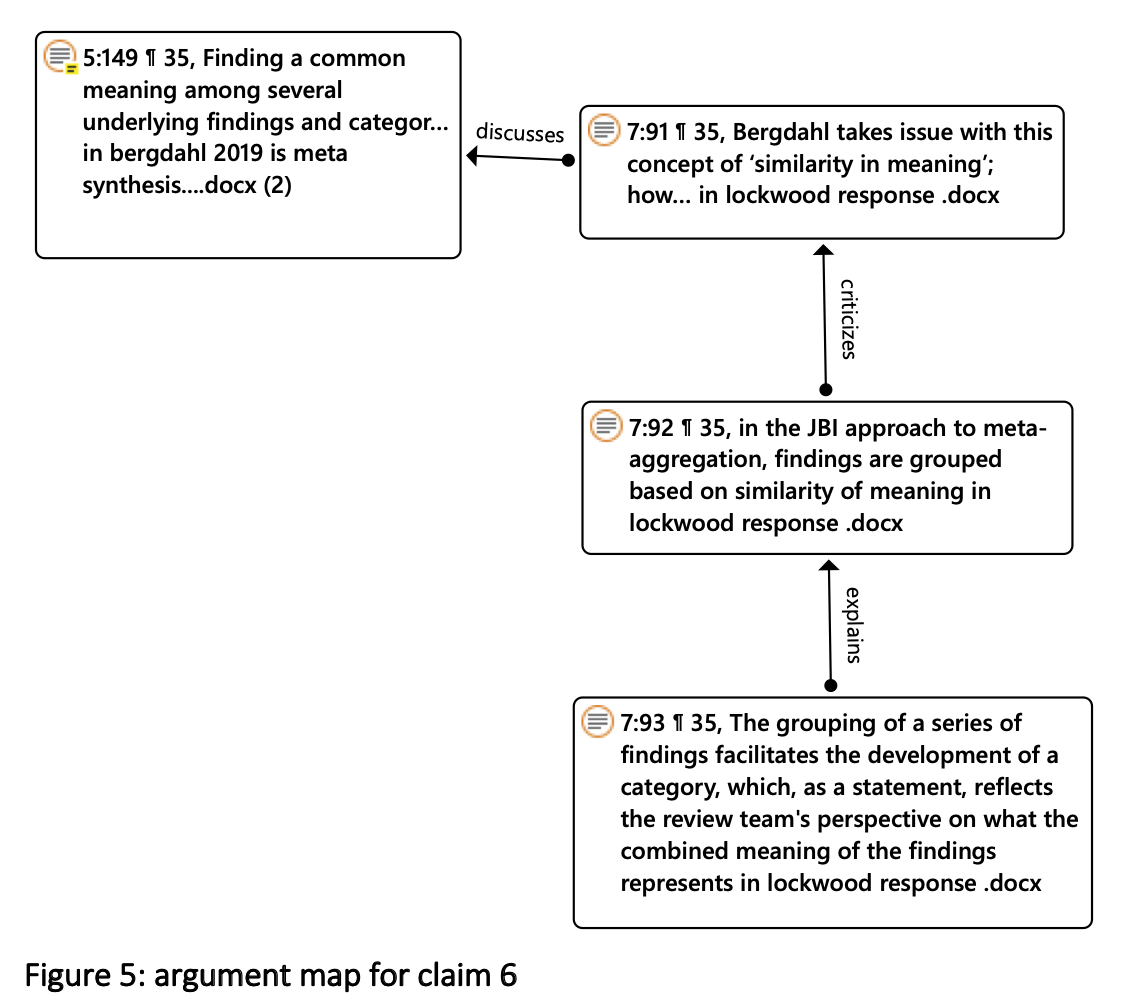

Supplement: Supplementary file 1 — Supporting information. [file NIN-29-e12465-s001.docx]
